# Supplementary material for: Is health literacy of family carers associated with carer burden, quality of life, and time spent on informal care for older persons living with dementia?
Source: PLoS One. 2020 Nov 20;15(11):e0241982. doi: 10.1371/journal.pone.0241982 (PMC7678960; doi:10.1371/journal.pone.0241982)
Supplement: S3 File — (PDF) [file pone.0241982.s003.pdf]

HLS-Q12

On a scale from very difficult to very easy, how easy would you say it is to:

		very difficult	difficult	easy	very easy	don't know
1	find information on treatments of illnesses that concern you? Q2	1	2	3	4	
2	understand what to do in a medical emergency? Q7	1	2	3	4	
3	judge the advantages and disadvantages of different treatment options? Q10	1	2	3	4	
4	follow the instructions on medication? Q14	1	2	3	4	
5	find information on how to manage mental health problems like stress or depression? Q18	1	2	3	4	
6	understand why you need health screenings (e.g. breast exam, blood sugar test, blood pressure)? Q23	1	2	3	4	
7	judge if the information in the media on health risks is reliable (TV, internet or other media)? Q28	1	2	3	4	
8	decide how you can protect yourself from illness based on advice from family and friends? Q30	1	2	3	4	
9	find information on healthy activities such as exercise, healthy food and nutrition? Q32	1	2	3	4	
10	understand information on food packaging? Q38	1	2	3	4	
11	judge which everyday behaviour is related to your health (drinking and eating habits, exercise etc.)? Q43	1	2	3	4	
12	make decisions to improve your health? Q44	1	2	3	4	

HC 1-4; DP 5-8; HP 9-12

Developed by Finbråten, Pettersen og Guttersrud (2017)[1] on basis of the European Health Literacy Survey Questionnaire (HLS-EU-Q47; HLS-EU Consortium, 2012)[2]

[1]published in: Finbråten, H. S., Wilde-Larsson, B., Nordström, G., Pettersen, K. S., Trollvik, A., & Guttersrud, Ø. (2018). Establishing the HLS-Q12 short version of the European Health Literacy Survey Questionnaire: latent trait analyses applying Rasch modelling and confirmatory factor analysis. *BMC Health Services Research, 18*(1), 506.

[2]HLS-EU Consortium. Comparative report of health literacy in eight EU member states. The European Health Literacy Survey HLS-EU. 2012.
