# Supplementary material for: Is health literacy of family carers associated with carer burden, quality of life, and time spent on informal care for older persons living with dementia?
Source: PLoS One. 2020 Nov 20;15(11):e0241982. doi: 10.1371/journal.pone.0241982 (PMC7678960; doi:10.1371/journal.pone.0241982)
Supplement: S4 File — (PDF) [file pone.0241982.s004.pdf]

1

Er du pårørende (familie, venn, nabo etc) til en person med aldersrelatert hukommelsessvikt eller mistenkt/påvist demenssykdom? **Ja** ☐ **Nei** ☐

Svarte du nei, trenger du ikke å fylle ut mer av undersøkelsen.

2

Hvilket av disse utsagnene beskriver best det daglige funksjonsnivået til personen med hukommelsessvikt/demens? Sett ett kryss for det du synes passer best.

Klarer seg selv i alle omgivelser, men er glemsom eller avbryter ofte aktiviteter i dagliglivet.. ☐

Fungerer uten rettledning i kjente omgivelser .... ☐

Trenger veiledning for å fungere selv i kjente omgivelser. Kan nyttiggjøre seg muntlige instruksjoner .... ☐

Trenger assistanse for å fungere. Klarer ikke å følge kun muntlige instruksjoner .... ☐

Er fysisk sprek, men trenger hjelp for å fungere. Kan ikke kommunisere muntlig på en meningsfull måte .... ☐

Sengeliggende eller sitter i en stol uten evne til å gå rundt, reagerer kun på berøring .... ☐

3

Mange bruker mye tid på å hjelpe og støtte personer med hukommelsessvikt/demens. Kan du anslå hvor mye tid du bruker på en typisk omsorgsdag?
En typisk omsorgsdag er en gjennomsnittlig dag hvor du hjelper vedkommende.

På en *typisk omsorgsdag*, hvor mye tid brukte du på å hjelpe personen med oppgaver som toalettbesøk, måltider, påkledning, stell, forflytning og bading? .... timer

I løpet av de siste 30 dager, hvor mange dager brukte du på å hjelpe personen med slike gjøremål? .... dager

På en *typisk omsorgsdag*, hvor mye tid brukte du på å hjelpe personen med oppgaver som innkjøp, matlaging, husarbeid, klesvask, hagearbeid, vedlikehold av bolig, medisinering og håndtering av økonomi? .... timer

I løpet av de siste 30 dager, hvor mange dager hjalp du personen med slike gjøremål?............ dager

På en *typisk omsorgsdag*, hvor mye tid per dag brukte du på å snakke med personen i telefonen? .... timer

I løpet av de siste 30 dager, hvor mange dager brukte på dette? .... dager

I løpet av de siste 30 dager, hvor mange ganger fulgte du personen til avtaler (legetime, tannlege, frisør, fotpleier, fysioterapi, dagsenter etc) .... ganger

Hvor lang tid brukte på dette pr gang? (i gjennomsnitt) .... timer

I løpet av den siste **uken**, hvor mye tid brute du på å prøve å få tak i helsepersonell, koordinere/omrokkere på avtaler eller søke etter informasjon om helsetjenester på vegne av personene med hukommelesessvikt/demens? (inkludert møter, telefontid, søk på internett osv) .... timer

4

Vi ønsker å vite i hvilken grad du opplever noen belastning ved å være pårørende til en person med hukommelsessvikt/demens. For hvert spørsmål, sett en ring rundt det tallet som passer best for deg fra 1=aldri/ingen, til 5=alltid/svært mye

	aldri/ingen<br>1	sjelden/litt<br>2	av og til/moderat<br>3	ofte/ganske mye<br>4	alltid/svært mye<br>5
Føler du noen gang at du ikke lenger holder ut?	1	2	3	4	5
Føler du noen gang at du trenger ferie/avkopling?	1	2	3	4	5
Blir du noen gang deprimert over den situasjonen du er i?	1	2	3	4	5
Går omsorgsarbeidet ut over din egen helse?	1	2	3	4	5
Er du redd det kan skje en ulykke med pasienten?	1	2	3	4	5
Føler du noen gang at det ikke finnes løsning på dine vanskeligheter?	1	2	3	4	5
Er det vanskelig for deg å kunne dra på ferie?	1	2	3	4	5
Hvor mye går omsorgen for pasienten ut over ditt sosiale liv?	1	2	3	4	5
Hvor mye er rutiner i hjemmet ditt blitt forandret på grunn av pasienten?	1	2	3	4	5
Er søvnen din forstyrret på grunn av pasienten?	1	2	3	4	5
Er din livskvalitet/livsstandard blitt redusert på grunn av pasienten?	1	2	3	4	5
Er du flau på vegne av pasienten?	1	2	3	4	5
Er du forhindret fra å ha gjester hjemme grunnet pasienten?	1	2	3	4	5
Blir du noen gang sliten og sur på pasienten?	1	2	3	4	5
Blir du noen gang frustrert (oppgitt) sammen med pasienten?	1	2	3	4	5

5

Du som er pårørende kan også ha egne helseplager.
Vi ønsker å kartlegge din helse slik du opplever den akkurat nå.

Under hver overskrift ber vi deg krysse av den ENE boksen som best beskriver helsen din I DAG.

GANGE

Jeg har ingen problemer med å gå omkring ❑
Jeg har litt problemer med å gå omkring ❑
Jeg har middels store problemer med å gå omkring ❑
Jeg har store problemer med å gå omkring ❑
Jeg er ute av stand til å gå omkring ❑

PERSONLIG STELL

Jeg har ingen problemer med å vaske meg eller kle meg ❑
Jeg har litt problemer med å vaske meg eller kle meg ❑
Jeg har middels store problemer med å vaske meg eller kle meg ❑
Jeg har store problemer med å vaske meg eller kle meg ❑
Jeg er ute av stand til å vaske meg eller kle meg ❑

VANLIGE GJØREMÅL *(f.eks. arbeid, studier, husarbeid, familie- eller fritidsaktiviteter)*

Jeg har ingen problemer med å utføre mine vanlige gjøremål ❑
Jeg har litt problemer med å utføre mine vanlige gjøremål ❑
Jeg har middels store problemer med å utføre mine vanlige gjøremål ❑
Jeg har store problemer med å utføre mine vanlige gjøremål ❑
Jeg er ute av stand til å utføre mine vanlige gjøremål ❑

SMERTER / UBEHAG

Jeg har verken smerter eller ubehag ❑
Jeg har litt smerter eller ubehag ❑
Jeg har middels sterke smerter eller ubehag ❑
Jeg har sterke smerter eller ubehag ❑
Jeg har svært sterke smerter eller ubehag ❑

ANGST / DEPRESJON

Jeg er verken engstelig eller deprimert ❑
Jeg er litt engstelig eller deprimert ❑
Jeg er middels engstelig eller deprimert ❑
Jeg er svært engstelig eller deprimert ❑
Jeg er ekstremt engstelig eller deprimert ❑

- Vi vil gjerne vite hvor god eller dårlig helsen din er I DAG.
- Denne skalaen er nummerert fra 0 til 100.
- 100 betyr den <u>beste</u> helsen du kan tenke deg.
  0 betyr den <u>dårligste</u> helsen du kan tenke deg.
- Sett en X på skalaen for å angi hvordan helsen din er I DAG.
- Skriv deretter tallet du merket av på skalaen inn i boksen nedenfor.

HELSEN DIN I DAG = ☐

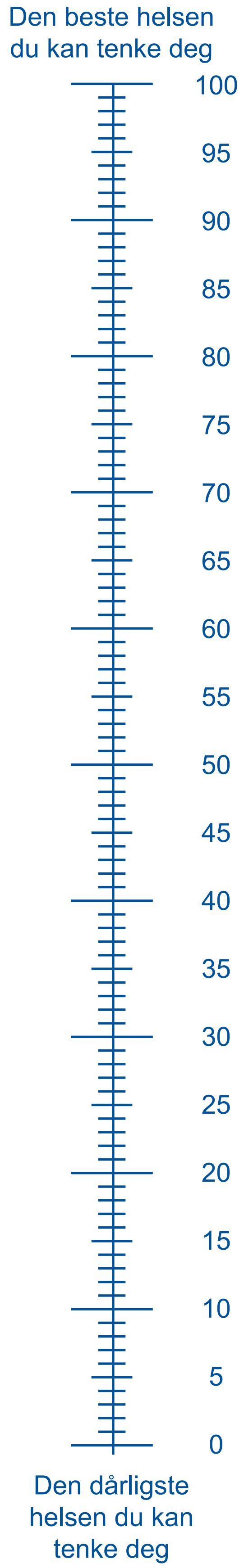

61316_Spørreskjema_pårørende_A4.indd 4
16.01.2019 09:11

6

Noen opplever det vanskelig å få den helsehjelpen de trenger. Vi vil kartlegge hvor lett eller vanskelig du synes det er å finne, vurdere og benytte helseinformasjon. Selv om ikke alle spørsmålene er relevante for deg er det fint om du svarer det som passer best.
Sett en ring rundt ett av tallene 1-6 på hver linje. 1=veldig lett, 6= veldig vanskelig

	veldig lett					veldig vanskelig
**Hvor lett/vanskelig er det for deg å:**	**1**	**2**	**3**	**4**	**5**	**6**
- finne opplysninger om behandlinger av sykdommer som gjelder deg?	1	2	3	4	5	6
- forstå hva som bør gjøres i en medisinsk akuttsituasjon?	1	2	3	4	5	6
- vurdere fordeler og ulemper ved ulike typer behandling?	1	2	3	4	5	6
- følge bruksanvisningene som er angitt på forpakningen til legemidler?	1	2	3	4	5	6
- finne informasjon om hvordan psykiske problemer som stress og depresjon kan takles?	1	2	3	4	5	6
- forstå hvorfor du har behov for generelle helseundersøkelser (f.eks. mammografi, måle blodsukkeret og blodtrykket)?	1	2	3	4	5	6
- vurdere om opplysninger som media gir om helserisiko er til å stole på (TV, internett eller andre medier)?	1	2	3	4	5	6
- avgjøre hvordan du kan unngå sykdom på bakgrunn av råd fra familie og venner?	1	2	3	4	5	6
- finne informasjon om sunne vaner, som mosjon, sunn mat og riktig ernæring?	1	2	3	4	5	6
- forstå informasjon på matemballasje?	1	2	3	4	5	6
- bedømme hvilke hverdagsvaner som har sammenheng med helsen din (spise- og drikkevaner, mosjon, osv.)?	1	2	3	4	5	6
- ta avgjørelser for å bedre helsen din?	1	2	3	4	5	6
- forstå medisinsk fagspråk og terminologi	1	2	3	4	5	6
- forstå hvilken informasjon som er relevant å gi til helsepersonell	1	2	3	4	5	6
- formidle helsetilstanden og dine behov til helsepersonell på en medisinsk forståelig og effektiv måte	1	2	3	4	5	6

7

Hvilket år er du født?....................

Er du født i Norge? Ja ☐ Nei ☐

Kjønn: Kvinne ☐ Mann ☐

Er den du er pårørende til, født i Norge? Ja ☐ Nei ☐

Hva er ditt **postnummer**.........................

Bor den du hjelper:

I egen bolig ☐ På institusjon ☐ Sammen med deg ☐

Hvem er den du hjelper? (sett ett kryss)

Ektefelle/samboer/partner ☐ Annen familie ☐ Annet ☐

Hva er den høyeste utdanningen du har fullført? (sett ett kryss)

Grunnskole ☐

Høgskole/universitet inntil 3 år ☐

Videregående skole/fagbrev/mesterbrev ☐

Mer enn 3 år på høgskole eller universitet ☐

Har du noen gang arbeidet som helsepersonell? Ja ☐ Nei ☐

Er du yrkesaktiv nå? (sett ett kryss)

Ja, jeg jobber vanligvis ....timer pr uke

Nei - jeg er pensjonist ☐

- jeg er uføretrygdet ☐

- jeg er ikke yrkesaktiv av annen årsak ☐

Har ditt rolle som pårørende ført til at du har: (sett ett kryss)

- arbeidet mer eller holdt deg lenger i lønnet arbeid Ja ☐ Nei ☐
- avsluttet yrkesaktiviteten tidligere Ja ☐ Nei ☐
- redusert stilling eller byttet til lavere lønnet arbeid Ja ☐ Nei ☐

Tusen takk for hjelpen!
