# Supplementary material for: Is health literacy of family carers associated with carer burden, quality of life, and time spent on informal care for older persons living with dementia?
Source: PLoS One. 2020 Nov 20;15(11):e0241982. doi: 10.1371/journal.pone.0241982 (PMC7678960; doi:10.1371/journal.pone.0241982)
Supplement: S5 File — (DOCX) [file pone.0241982.s005.docx]

Translation of the questionnaire

The Norwegian questionnaire, which is uploaded as supporting information, is consisting of already existing questionnaires which are translated to Norwegian. We will hearby give a translation of the survey. Are you a family carer (family, friend, neighbor) to a person with age-related cognitive decline, suspected dementia or diagnosed dementia? (yes/no)

1. (The Berger dementia Scale) Which of these statements best describe the daily function of the person living with cognitive decline/dementia? Please mark the one statement that you feel fits best.
   - Can function in any surroundings, but forgetfulness is often disruptive of daily activities
   - Can function without direction only in familiar surroundings
   - Needs direction to function even in familiar surroundings but can respond appropriately to instruction
   - Needs assistance to function, cannot respond to direction alone
   - Remains ambulatory, needs assistance to function, but cannot communicate verbally in a meaningful fashion
   - Bedridden or confined to a chair and responds only to tactile stimuli
2. (Modified RUD questionnaire) (the translation is made by the first author. Validated English versions will probably vary slightly. In the Norwegian survey we used a validated version in Norwegian)
   - On a typical care day, how much time did you spend on helping a person with tasks like going to the toilet, meals, dressing, grooming, moving around and bathing? (answer in number of hours)
   - During the last 30 days, how many days did you spent on caring out these tasks? (answer number of days)
   - On a typical care day, how much time did you spend on helping with tasks like shopping, cooking, housework, laundry, gardening, maintenance of house, helping with medication and handling of banking? (answer in number of hours)
   - During the last 30 days, how many days did you help with such tasks? (answer in number of days)
   - On a typical care day, how much time did you spend talking to the care-recipient on the phone? (answers in number of hours)
   - During the last 30 days, how many days did you do this? (answer in number of days)
   - During the last 30 days, how many times did you go with the care-recipient to appointments (doctor’s appointment, dentist, hairdresser, pedicure, physiotherapist, day activity center etc)? (answer in number of times)
   - How much time did you spend each time, on average? (answer in number of hours)
   - During the last week, how much time did you spend trying to reach healthcare personnel, coordinating/changing appointments or searching for information about health services on behalf of the person living with dementia? (answer in number of hours)
3. (The Relative Stress Scale)
   - 1. Do you ever feel you can no longer cope with the situation?
   - 2. Do you ever feel you need a break?
   - 3. Do you ever get depressed by the situation?
   - 4. Has your own health suffered at all?
   - 5. Do you worry about accidents happening to the patient?
   - 6. Do you ever feel that there will be no end to the problem?
   - 7. Do you find it difficult to get away on holiday?
   - 8. How much have your social life been affected?
   - 9. How much has the household routine been upset?
   - 10. Is your sleep interrupted by the patient?
   - 11. Has your standard of living been reduced?
   - 12. Do you ever feel embarrassed by the patient?
   - 13. Are you at all prevented from having visitors?
   - 14. Do you ever get cross or angry with the patient?
   - 15. Do you ever feel frustrated at times with the patient?
4. (EQ-5D-5L) See the picture below of the english version of EQ-5D-5L


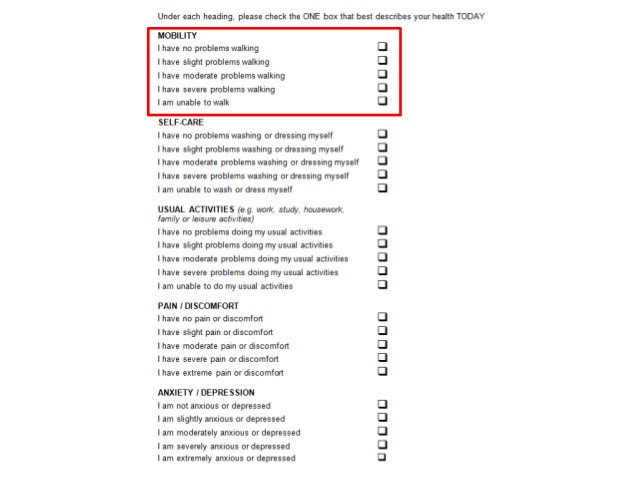

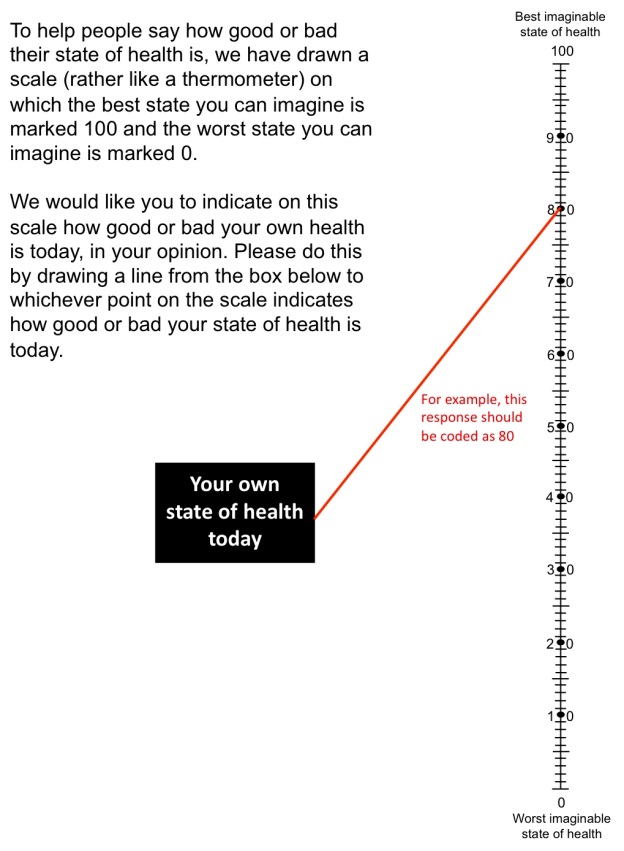


1. English version of the Health Literacy Scale (HLS-Q12). Please see attached file named HLS_Q12_English2
   - Three additional questions were added to the end of the HLS-N-Q12. The three questions were not use in analysis and not reported in the paper.
     1. –understanding medical language and terminology
     2. Understanding which information is relevant for healthcare personnel
     3. Communicate your health state and your needs to healthcare personnel in a medically understandable and effective manner.
2. Additional background questions:
   - Which year are you born
   - Are you female/male
   - Are you born in Norway (yes/no)
   - Is the care-recipient born in Norway (yes/no)
   - What is your postal code
   - Is the care-recipient living independently/in institution/shared household with you
   - What is your relationship to the care recipient (partner or spouse/ other family/other)
   - What is your highest completed education
     1. primary school (9 years)
     2. secondary school (12 years)
     3. Up to three years of university education
     4. more than three years of university education.
   - Have you worked as health personnel (yes/no)
   - Are you working (paid work away from home) (yes, I usually work ___hours per week/ no, I am retired/ no, I am receiving disability benefits/ no, I am not working for other reasons)
   - Have your role as family carer led to (more or prolonged work/early retirement from work/reduced work hours or lower paid work)
